# Validation of Rapid Interactive Screening Test for Autism in Toddlers Using Autism Diagnostic Observation Schedule™ Second Edition in Children at High-Risk for Autism Spectrum Disorder

**DOI:** 10.3389/fpsyt.2021.737890

**Published:** 2021-10-01

**Authors:** Xue-Jun Kong, Hannah Tayla Sherman, Ruiyi Tian, Madelyn Koh, Siyu Liu, Alice Chukun Li, William S. Stone

**Affiliations:** ^1^Athinoula A. Martinos Center for Biomedical Imaging, Massachusetts General Hospital, Charlestown, MA, United States; ^2^Department of Psychiatry, Massachusetts Mental Health Center, Beth Israel Deaconess Medical Center, Boston, MA, United States

**Keywords:** early screening, Asian, social affect (SA), restricted and repetitive behaviors (RRB), autism spectrum disorder (ASD)

## Abstract

The Rapid Interactive screening Test for Autism in Toddlers (RITA-T) is a fast and inexpensive early screening measure for autism spectrum disorder (ASD) that was tested previously in children 18–36 months-old; the current validation study compared the RITA-T with the Autism Diagnostic Observation Schedule™ Second Edition (ADOS-2). The hypothesis is to validate the RITA-T with comparison to the ADOS-2. Thirty-five individuals (18–84 months-old) identified as at risk for ASD received the RITA-T and the ADOS-2 during a single visit. Participants were split into two age groups and both whole-group and sub-group data analysis were conducted. With all participants, RITA-T scores correlated significantly with ADOS-2 total scores (*P* < 0.001), social affect (SA) sub-scores (*P* < 0.001), and restrictive and repetitive behavior (RRB) sub-scores (*P* < 0.05). Similarly, ADOS-2 total and SA scores were significantly correlated in both age groups, while the RRB sub-score was only significant in females (*P* < 0.05). Lastly, correlations using subgroups based on ethnicity were only significant in the minority (“Other”) group for ADOS-2 total scores and in the Asian group for SA sub-scores (*P* < 0.05). Our receiver operating characteristic analysis showed that the optimal cut-off score of the RITA-T was consistently at 14, with a sensitivity of 81% and a specificity of 89% in the combined age group with the ADOS-2 and with a sensitivity 74% and specificity 50% with the DSM-5; The area under the curve was 0.84 (95%CI: 0.69–0.99) for ASD classified by ADOS-2 and 0.89 (95%CI: 0.79–0.99) for ASD diagnosed by DSM-5. The RITA-T performed similarly to the ADOS-2 when both were administered in a single visit. Significant correlations between the measures help validate the potential usefulness of the RITA-T as a rapid early screening measure of ASD. This study helps to show that the RITA-T may be used in a larger age range than originally reported and in different ethnic groups. The study involves human participants and was reviewed and approved by the Institutional Review Board (IRB) of Massachusetts General Hospital (MGH, 2017P0000857).

## Introduction

Early diagnosis and intervention significantly impact the prognosis of individuals with Autism Spectrum Disorder (ASD) and underscores the importance of easily applied early detection and screening tools (1–7). Standard evaluation methods for ASD, such as Autism Diagnostic Observation Schedule™ (ADOS) and *Autism Diagnostic Interview-Revised* (ADI-R), have been developed to provide an official diagnosis (8, 9), but these standard methods are often lengthy, difficult, and costly. Shortages in resources for such evaluations may thus delay diagnosis and treatment (10, 11). The mean age of diagnosis is still 4–5 years old worldwide despite recent advances and efforts (7). In addition, the difficulties posed by standard evaluation methods are even greater among minority groups (e.g., African American, Hispanic, Asians, and etc.) in the USA due to language and cultural barriers, including a bias against mental diseases or conditions (12–15). To improve this situation, easier, and faster alternative methods have been proposed and applied with various feasibilities and sensitivities (6, 16). As a group, these methods need more study before they can be recommended with confidence to families, educators, and healthcare providers.

The Rapid Interactive screening Test for Autism in Toddlers (RITA-T) is one of the alternative screening methods that may have the potential for widespread use due to its easy administration and rapid interpretation. The RITA-T screener could better stratify the likelihood of ASD, thereby leading to more focused evaluations and faster diagnoses. The RITA-T is intended to function as an ASD screening test (a “level 2 screener”), but not as a general population screener, such as the Modified Checklist for Autism in Toddlers (M-CHAT; a “level 1 screener”). Relative to M-CHAT, which is a freely accessible questionnaire that is completed by either a parent or a caregiver, the RITA-T is a level 2 screener that can be administered by professionals working with young children, including teachers, childcare workers, pediatricians, nurses, family physicians, and pediatric behavioral health professionals. As such, the RITA-T streamlines the access of screening for those who need further evaluation directing a formal diagnosis for ASD. Training for the RITA-T is 3 h. This behavioral evaluation includes 9 interactive games that can be completed in 10 min. The first published paper using the RITA-T compared it to clinical diagnosis (*n* = 60) and reported good sensitivity (100%) and encouraging specificity (84%) (16). A larger study (*n* = 239) recently reported good sensitivity (97%) but lower specificity (71%) with the same cut off score of 14 (17). However, the RITA-T score has not yet been evaluated in the context of the ADOS-2 sub-scores, which includes social affect (SA) and restricted and repetitive behavior (RRB). This is significant because the diagnosis of ASD requires both SA deficits and RRB. If someone only has the SA deficit, for example, but not the RRB deficit, the diagnostic criteria for ASD are not met. The RITA-T is designed to evaluate only SA without assessment or scoring of RRB, which might explain the relatively lower specificity in both published studies. In addition, the RITA-T has also not been assessed across different racial or extended age groups beyond toddlers (16, 17). In this study, we aim to fill these gaps by further evaluating the validity of the RITA-T through a direct comparison with ADOS-2, as part of the larger goal of expanding available resources for early detection of ASD, particularly extending the age group to and beyond preschoolers as the current median age of ASD diagnosis is reported to be 51 months old (7, 18, 19).

## Materials and Methods

### Participants

Thirty-five individuals aged 18–84 months old toddlers and preschoolers (mean = 3.53 years, standard deviation = 1.72 years) participated in this study. During participant recruitment, we reached out to different ethnic groups to encourage a more ethically heterogenous study cohort. The enrolled study participants included 15 White (43%), 11 Asian (31%), and 9 other race (26%) subjects. Asian is defined here as an individual who is a descendant of an individual who was born in Southeast Asia, the Far east or the Indian subcontinent. Twenty-three males and twelve females participated in this study. Sex is defined by sex chromosomes composition: males have XY chromosomes, and females have XX chromosomes. Individuals were recruited through clinical care clinics and online recruitment sites. Most participants came from Massachusetts and its surrounding states. All participants were included after being identified as high-risk for ASD by clinicians or caregivers. The high-risk status was confirmed through a phone screening prior to enrollment. Inclusion criteria included one or more of the following: (1) at least one sibling with a clinical diagnosis of ASD; (2) a caregiver or clinician indicated concerns about the child's development of social interaction, play, or other behaviors; and/or (3) the individual scored in the positive range on the M-CHAT. Exclusion criteria included major congenital or genetic disorders or diseases, or behavioral problems that would cause substantial additional stress for the family and/or the child during testing. Individuals with a previous diagnosis of ASD were included, but the examiner was not informed of the diagnosis.

### Assessment Instruments and Protocols

The present study involves human participants and was reviewed and approved by the Institutional Review Board (IRB) of Massachusetts General Hospital (MGH, 2017P0000857). Written informed consent to participate in this study was provided by the participant's legal guardian. RITA-T is a level-2 ASD screening test which includes 9 interactive games (phone block, phone tease, block vision, object constancy, color constancy, face vs. object, rapid joint attention, reaction to emotion, and self-recognition) that takes about 10 min to complete by trained and certified staff. The training and certification only need 3 h to finish and applies to entry level clinical and research staff. The RITA-T focuses on five major areas: joint attention, social awareness, human agency, self-recognition, and fundamental cognitive skills. The total score ranges from 0 to 30 (16). Responses were scored during test administration.

The ADOS-2 is considered the “gold standard” in diagnosis and is a semi-structured, standardized assessment of social interactions, language and communication, repetitive, restricted patterns of behavior and interest, and play and imagination (20–22). It contains five modules that are differentiated by participant's developmental and language levels (Module T, 1, 2, 3, and 4). Every ADOS-2 module ends with a diagnostic algorithm(s) that consists of selected items that have been chosen to maximize diagnostic sensitivity and specificity. In this study, the ADOS-2 was administered by professionally trained investigators, in consultation with a certified ADOS trainer, as needed. Following the standardized algorithm of corresponding modules, the composite score, SA, and RRB sub-scores were all recorded for each subject on a commercially available standard score booklet and scored right after the visit. The RITA-T and the ADOS-2 were both administered in one study visit in a randomized order. The ADOS-2 was administered by two different professionally trained administrators and the RITA-T was administered by three different professionally trained administrators. The overall evaluation time is around 1 h. Diagnostic and Statistical Manual, Fifth Edition (DSM-5) diagnostic criteria (23) were used by trained clinicians to diagnose ASD based on clinical observation, independent of the ADOS-2 and RITA-T scores.

### Statistics

We first calculated descriptive statistics for demographic variables including age, sex, and race, and for RITA-T score, ADOS-2 total, and ADOS-2 sub-scores. We also examined distributions of ASD vs. non-ASD subjects categorized by both ADOS-2 and DSM-5.

Since scores in the ADOS-2 from module T and 1 & 2 are not directly comparable, ADOS-2 scores (total score, sub-score SA, and RRB scores) were converted to calibrated severity scores (CSS), including total CSS, SA CSS, and RRB CSS using a previously published and well-recognized conversion method, and were checked for normality via the Shapiro-Wilk test (24–27). Correlations of RITA-T scores with ADOS-2 CSS and the sub-score CSSs were calculated using linear regression via the Kendall rank correlation and the correlation coefficients were compared across age, race, and sex groups.

Receiver operating characteristic (ROC) curve analysis was performed to validate the predictive performance of RITA-T scores by determining its optimal cut-off score compared with that of ADOS-2 and DSM-5. The ROC curves were also compared between age groups (18~36 mo.; 37–84 mo.). Sensitivity, specificity, positive predictive value (PPV), and negative predictive value (NPV) of the RITA-T score in predicting the final diagnosis were computed for each age group and the combined group, and the cut-off scores with the optimal sensitivity and specificity were determined for the entire group and for each age group.

## Results

### Demographic Features and ADOS-2 Score Compositions of the Participants

Table 1 shows that 40% of the subjects were 18–36 months old and 60% were 37–84 months old; among subjects aged 37–84 months old, 13 subjects were of ages 3.08–5 years old and 8 subjects were of ages 5–7 years old. Sixty-six percent were male and 34% were female. Thirty-one percent were Asian, 43% were White and 26% were in the others category. Among all subjects, 29 out of 35 (82.8%) were classified with ASD by ADOS-2, and 25 out of 35 (71.4%) were diagnosed with ASD using DSM-5 diagnostic criteria. Of those 18–36 months old, 11 out of 14 (78.5%) were classified with ASD by ADOS-2 while in the 37 to 84-month-old group, 18 out of 21 (85.7%) were classified with ASD by the ADOS-2 and 15 out of 21 (71.4%) were diagnosed using DSM-5. Subjects classified with ASD the by the ADOS-2 included 9 out 11 Asians (81.8%), 11 out of 15 Whites (83.3%), and 9 out of 9 others (100%). They also included 21 out 23 males (91.3%) and 8 out of 12 females (66.6%). Among all the participants in this study, 12 of them had a sibling with ASD, 9 of them had positive M-CHAT test, and 33 of them have had concerns from caregivers.

**Table 1:** Demographic features of the participants by age, sex, and race.

**Age**		**18–36 months**		**37–84 months**		**Total**	
Sex	Male	9	64%	14	67%	23	66%
	Female	5	36%	7	33%	12	34%
Race	White	5	36%	10	48%	15	43%
	Asian	3	21%	8	38%	11	31%
	Others	6	42%	3	15%	9	26%
Total		14	40%	21	60%	35	100%

### Correlation of RITA-T and ADOS-2 Total Scores/Sub-scores by Age, Race, and Sex

**Figure 1:**
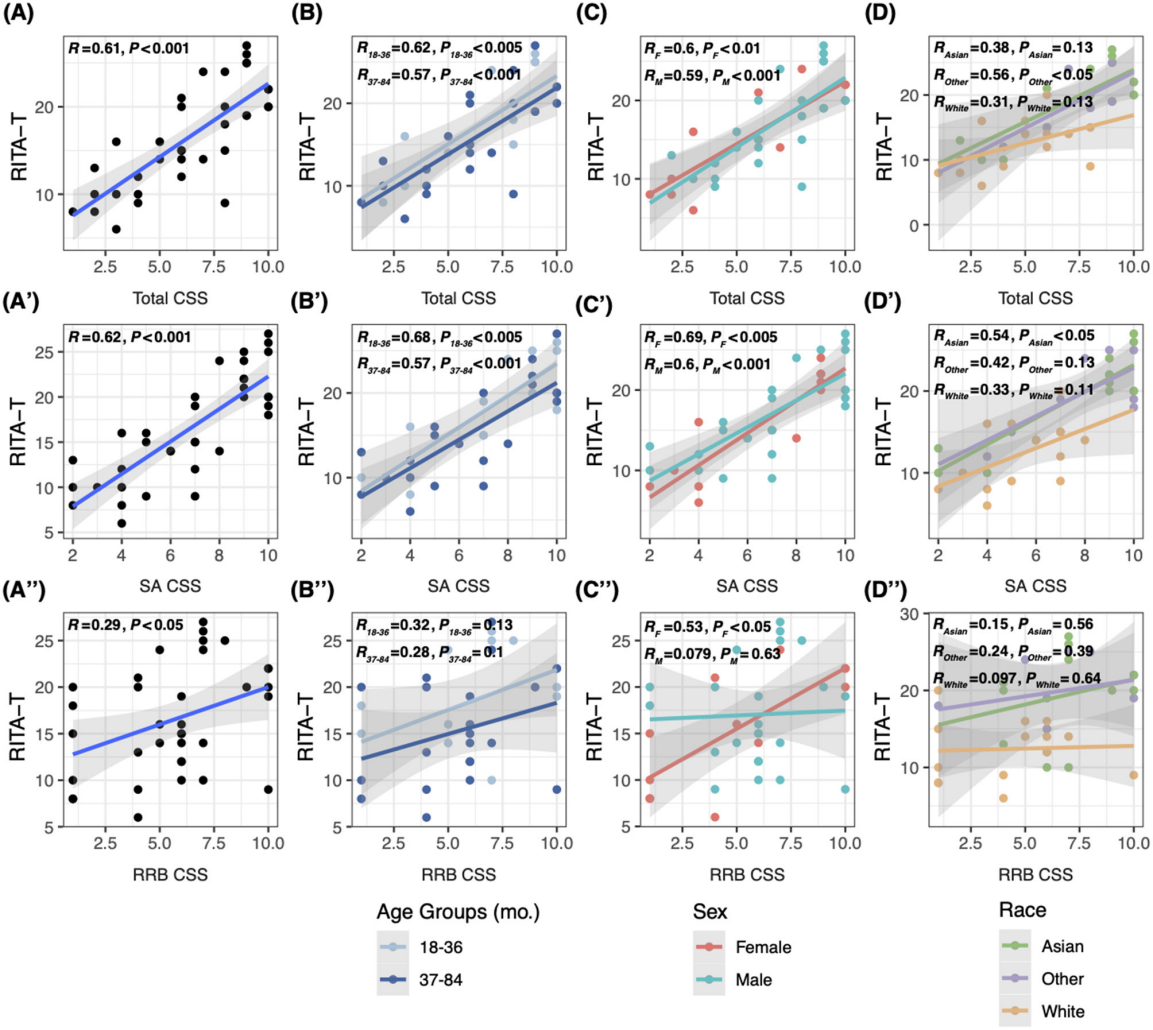
Linear regression model comparing correlations between RITA-T score and ADOS-2 CSS, total and sub-scores. **(A–A′′)** Correlations between scores of the RITA-T and ADOS-2 total CSS, SA CSS and RRB CSS in all subjects; **(B–B′′)** correlations between scores of the RITA-T and ADOS-2 in 18–36 and 37–84 month age subgroups; **(C–C′′)** correlations between scores of the RITA-T and ADOS-2 in male and female sex subgroups; **(D–D′′)** correlations between scores of the RITA-T and ADOS-2 in race subgroups.

Linear regression was used to evaluate correlations of RITA-T score with ADOS-2 total CSS, sub-scores SA CSS and RRB CSS on all subjects and in both age, race, and sex groups. The RITA-T was found to be significantly positively correlated with ADOS-2 total CSS (*P* < 0.001, *R* = 0.61), SA CSS (*P* < 0.001, *R* = 0.62), and RRB CSS (*P* < 0.05, *R* = 0.29, Figures 1A–A′′). Additionally, the RITA-T was significantly positively correlated with total CSS (*P*_18–36_ < 0.005, *R*_18–36_ = 0.52, *P*_37–84_ < 0.001, *R*_37–84_ = 0.57) and SA CSS (*P*_18–36_ < 0.005, *R*_18–36_ = 0.68, *P*_37–84_ < 0.001, *R*_37–84_ = 0.57), while RRB (*P*_18–36_ = 0.13, *R*_18–36_ = 0.32, *P*_37–84_ = 0.01, *R*_37–84_ = 0.28) did not show any significant correlation (Figures 1B–B′′). For race subgroups, RITA-T scores are significantly positively correlated with ADOS-2 total CSS among the minority (“Other”) subgroup (*P* < 0.05, *R* = 0.56, Figure 1D) and with SA CSS in the Asian subgroup (*P* < 0.05, *R* = 0.54, Figure 1D′), while other subgroups based on ethnicity did not show statistical significance in correlations with ADOS-2 total CSS, SA CSS, and RRB CSS (Figures 1D–D′′). In subgroups based on sex, both males and females demonstrated significant positive correlations with total CSS (*P*_*M*_ < 0.001, *R*_M_ = 0.59, *P*_*F*_ < 0.01, *R*_F_ = 0.6, Figure 1C) and SA CSS (*P* < 0.005, *R* = 0.69, Figure 1C′). However, correlations between RITA-T scores and RRB CSS are significant among females (*P* < 0.05, *R* = 0.53, Figure 1C′′), but not males (*P* = 0.63, *R* = 0.079).

### Correlation of RITA-T and ADOS-2/DSM-5 Classification/Diagnosis

Among all 35 subjects, 6 (17.1%) are not classified with ASD by ADOS-2 and 10 (28.6%) are not diagnosed/classified with ASD by DSM-5. RITA-T's sensitivity, specificity, PPV and NPV compared across ADOS-2 and DSM-5 for children 18–84 months old are shown in Supplementary Table 1. The cut-off score with most optimal sensitivity and specificity combination is 14 in this age group for ADOS-2 (sensitivity 81%, specificity 89%, PPV 96%, NPV 57%) and is also 14 for DSM-5 (sensitivity 74%, specificity 50%, PPV 74%, NPV 50%).

**Figure 2:**
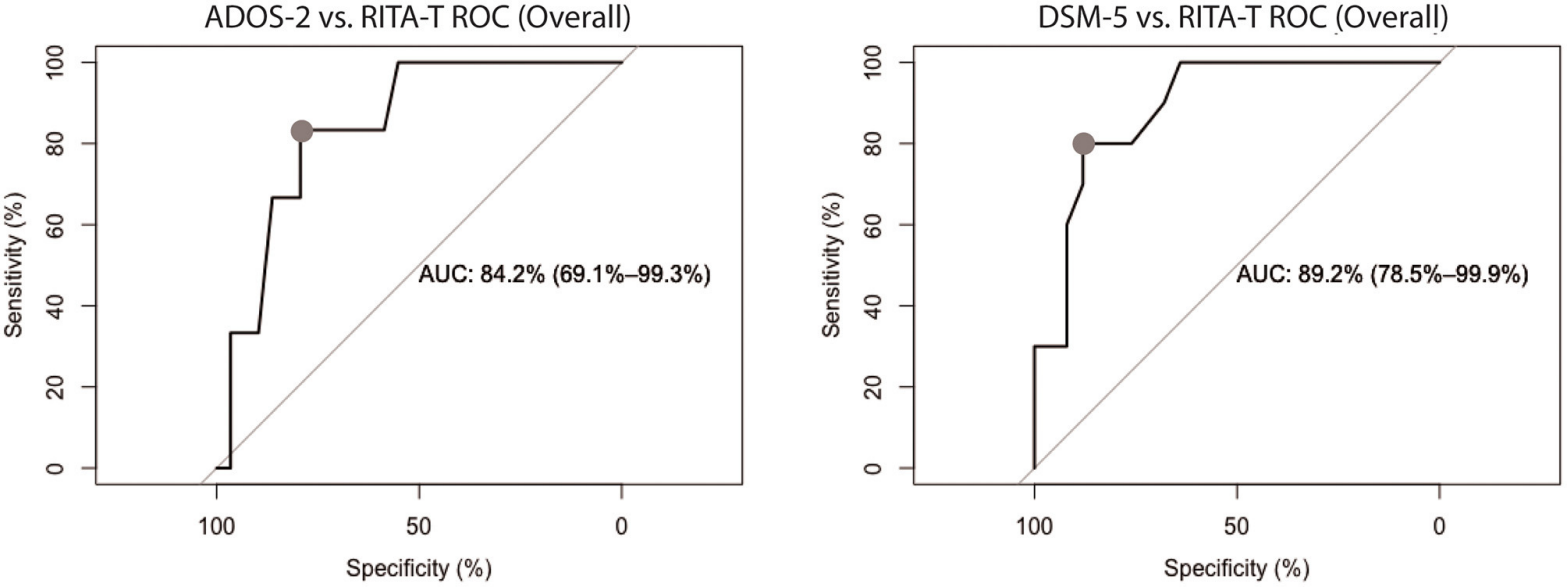
ROC curves of RITA-T in classifying ASD diagnosed by ADOS-2 and DSM-5 in 18 to 84-month old group, separately. The classification demonstrated a RITA-T cut-off score of 14 (dot) with higher optimal sensitivity and specificity. ADOS-2 ROC curve (Left) showed an AUC of 0.842 (95%CI: 0.691–0.993) while DSM-5 ROC curve (Right) indicated an AUC of 0.892 (95% CI: 0.785–0.999).

ROC curves for comparing RITA-T with ADOS-2 and RITA-T with DSM-5 are shown separately in Figure 2. The ADOS ROC curve showed an area under the curve (AUC) of 0.842 (95%CI: 0.691–0.993), while DSM-5 ROC curve indicated an AUC of 0.892 (95% CI: 0.785–0.999).

**Figure 3:**
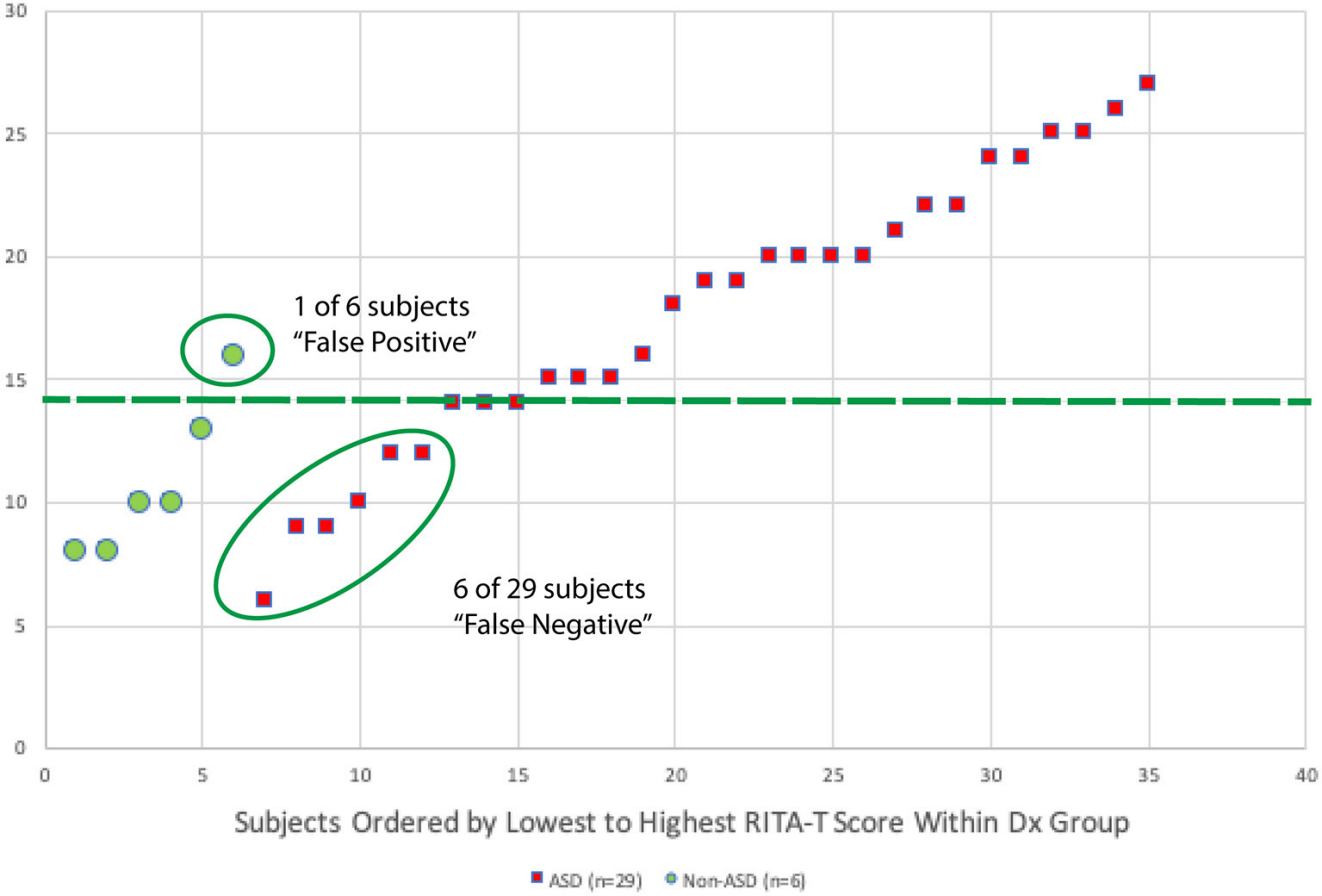
A scatter plot with distribution for RITA-T scores of each participant is shown in this figure. Diagnosis of ADOS-2 is used to separate ASD and non-ASD groups. With 14 as cut-off score (≥14 for ASD), a total of 1 out of 6 was false positive and a total of 6 out of 29 were false negative. All 6 of the false positive cases are subjects in age group 37–84 months.

A scatter plot with the distribution of RITA-T scores is shown in Figure 3. Classification of ADOS-2 was used to separate ASD and non-ASD groups. With 14 as the cut-off score (≥14 for ASD), a total of only 1 out of 6 was false positive and a total of 6 out of 29 were false negative. All 6 of the false negative cases are subjects were in the 37 to 84-month-old age group.

The true positive and false negative analyses (Table 2) showed significant differences in RITA-T (*P* < 0.0001), ADOS-2 total score (*P* < 0.005), and ADOS-2 sub-score SA (*P* < 0.001), but did not show a significant difference in the ADOS-2 sub-score RRB (*P* = 0.429).

**Table 2:** Comparison of ADOS-2 CSS and RITA-T scores between true positive subjects and false negative subjects.

**Groups**	**RITA-T**	**ADOS-2 CSS**		
	**Total**	**Total**	**SA**	**RRB**
True positive (mean ± SD)	19.78 ± 4.16	7.83 ± 1.67	8.26 ± 1.68	6.22 ± 2.75
False negative (mean ± SD)	9.67 ± 2.25	4.83 ± 1.83	5.17 ± 1.47	6.00 ± 2.19
*T*-Test (*p*-value)	<0.0001	0.004	0.0008	0.429

ROC analysis was performed twice using ADOS-2 and DSM-5 diagnosis/classification independently as golden standard to determine the RITA-T cut-off score of 14. RITA-T's sensitivity, specificity, PPV, and NPV compared cross ADOS-2 and DSM-5 for those 18–84 months old is shown in Supplementary Table 1. The cut-off score with most optimal sensitivity and specificity combination is 14 in this age group for ADOS-2 (sensitivity 81%, specificity 89%, PPV 96%, NPV 57%) and 14 for DSM-5 (sensitivity 74%, specificity 50%, PPV 74%, NPV 50%).

Based on the distribution of RITA-T scores for each participant, classification of ADOS-2 is used to classify ASD and non-ASD groups. With 14 as cut-off score (≥14 for ASD), a total of 1 out of 6 was false positive and a total of 6 out of 29 were false negative. All 6 of the false positive cases are subjects in age group 37–84 months.

As presented in Table 2 for true positive subjects and false negative subjects, mean and standard variance for ADOS-2 CSS and RITA-T score are calculated. *T*-test is performed to compare the difference between the two groups. True positive and false negative groups have significant difference in ADOS-2 SA CSS, total CSS, and RITA-T score, but have no significant difference in ADOS-2 RRB CSS.

## Discussion

This study compared performance on the RITA-T and ADOS-2 to further validate the RITA-T as a screening tool for ASD. ADOS-2 requires comprehensive training and strict certification, and DSM-5 diagnosis could only be done by licensed professionals or specialists. The limited resources and availabilities of these providers are unable to meet the rapidly increasing demands, which could only delay the diagnosis. Found to be correlated with ADOS-2 scores, the RITA-T has its value in further stratifying those individuals at high-risk of ASD, which could allow them to initiate the service before a formal diagnosis could be achieved. Our findings demonstrated that RITA-T scores correlated significantly with the ADOS-2 total score and both the SA and RRB sub-scores in the study subjects, but not with the RRB sub-score in age-, sex-, and race-based subgroups. The RITA-T also showed good sensitivity (81%) and specificity (89%) with a cutoff score of 14. When the RITA-T is compared to the secondary outcome of DSM-5 at the same cut off score of 14 the sensitivity is 74% and the specificity is 50%. These results add to the validation of the RITA-T as a rapid screening measure for ASD in children.

Our results showed significant associations between the RITA-T and the ADOS-2 in both the 18–36 and the 37 to 84-month-old groups, which indicates the possibility of extending the age range in which these relationships have been studied (16, 17). This may also extend the validity of the RITA-T to an older age group, which is notable because despite ongoing efforts at early detection, ASD diagnosis is usually delayed to a median of 51 months old (7, 18, 19). Furthermore, one recent study showed, that only 44% of children who were diagnosed with ASD received an evaluation by 36 months of age (18). Despite on-going efforts in reducing the mean age of diagnosis, we have observed minimal changes in the average age of diagnosis. By extending the RITA-T age range, we are hopeful that it can further cover these older children who may not have gotten a diagnosis at a younger age. Importantly, RITA-T is still suitable for those chronologically older children with younger development age such as all subjects aged <4 years old in this study. The benefit of using the RITA-T is that anyone can be trained to administer it, including teachers and healthcare provider; this further suggests that a primary healthcare provider could administer it during a routine visit if warranted, as the training time required for its certified administration is only 3 h. This could allow an easy access to bridge for earlier diagnoses and interventions, which has been previously shown to improve outcomes of ASD individuals (7, 18, 19). Therefore, the RITA-T may be a particularly useful screening measure for children in the 37 to 84-month-old age range.

We also found similar relationships between RITA-T and ADOS-2 scores in different ethnic groups. Notably, correlations between RITA-T and ADOS-2 total was uniquely significant in the minority group (denoted as “Other”) whereas SA sub-scores were uniquely significant in the Asian subgroup but not in the other nor White groups. This is notable because, in addition to delays in ASD noted above, additional delays in diagnosis are common in minority groups (12–15). Several factors likely contribute to these delays (e.g., low socioeconomic status, cultural/language barriers, and shortage of accessible therapeutic resources or diagnostic testing, including the ADOS). Moreover, foreign language versions of ADOS-2, including Chinese, have not been validated formally (28). These findings suggest the RITA-T may be particularly useful in minority groups.

Consistent with our findings concerning age and race, our findings concerning sex showed that both male and female subjects showed strong correlations between RITA-T scores and the ADOS-2 total score and the SA sub-scores. One sex-related difference did emerge. While male subjects did not show significant correlations with the ADOS-2 RRB sub-scores, female subjects did. Since the RITA-T does not include RRB assessment, this may reflect a false positive finding. However, the finding may also provide insight into how sex-related differences in autism overlap with the assessment of autism. First, the male to female ratio of ASD is up to 4.3:1 (18, 19). It was about 2:1 in the current study, which makes the female group smaller (*n* = 12), less reliable and more subject to the influence of extreme scores. Second, females may, on average, show higher social and linguistic skills and fewer atypical repetitive behaviors (29). RRB behaviors are typically more severe in males than females (19, 30, 31), though at least one study reported that the differences might be small (32). Related to this point, sex differences have been reported in subcategories of RRB domains, with female showing more insistence on sameness and compulsivity, and males showing more stereotyped and restrictive behaviors (33). It is thus possible in the present study that mild, more restricted or atypical RRB behaviors in females showed enough overlap with non-RRB ADOS-2 items to contribute to a significant RITA-T—ADOS-2 RRB correlation, even in the absence of actual RITA-T RRB items.

Several limitations should be noted. First, the number of subjects in the study was modest (*n* = 35), which limits the effects we were able to detect. Future investigation will require larger sample size to officially validate these findings. Related to this point, and second, the small number of subjects allowed for the specific assessment of white and Asian subjects, but African-American, Hispanic, and multiracial subjects were combined in an “other” category. Given the potential effectiveness of the RITA-T in minority subjects, it will be important to recruit enough African-American and Hispanic subjects in future studies to study them in separate groups. Third, although the RITA-T was well-aligned with the ADOS-2 total score and SA sub-scores, it did not contain items related to the RRB sub-score and thus, generally did not correlate significantly with that sub-score. This reflects a design weakness in the RITA-T that will hopefully be corrected in future revisions. Fourth, the same group of examiners administered the screens and diagnostic measures and were not blinded to the findings. Consequently, we cannot rule out the possibility that rating and diagnostic decisions were fully independent of each other, although the scoring was usually done afterwards by the main examiner with inputs from other co-examiners and observers of the tests, review of video and discussion to resolve scoring agreements. Fifth, the RITA-T was administered before the ADOS-2, except when siblings came in at the same time (roughly 30% of the time), when the order was reversed for just one of them. It is thus possible that performance on the ADOS-2 was influenced by performance on the RITA-T (although this influence could be small because the content of the tests do not overlap and the RITA-T is a short test). Future investigations will counterbalance test order and apply the order consistently to avoid performance variability related to order of administration. Sixth, we did not correct statistically for familial influences on performance, though its low frequency reduced the likelihood it altered outcome measures.

Despite the limitations, these findings provide encouraging evidence for validation of the RITA-T as a rapid and useful screening tool for ASD in both toddlers and those chronologically older children with developmental ages <4 years old. Compared to existing, less specific measures such as the M-CHAT, the RITA-T may provide a more useful way to stratify risk for ASD and can thus refer children to the most appropriate diagnostic procedures and bridge the subsequent interventions.

## Data Availability Statement

The data that support the findings of this study are available on request from the corresponding author, X-JK.

## Ethics Statement

The studies involving human participants were reviewed and approved by Massachusetts General Hospital IRB. Written informed consent to participate in this study was provided by the participants' legal guardian/next of kin.

## Author Contributions

X-JK was the PI of the study, conceived the concept, designed and led the study and data analysis, drafted the first version, and revised the manuscript. X-JK, HS, SL, and MK contributed to data collections, data management, and analysis. RT contributed to data analysis, create tables, figures, and legend, and the literature search. HS contributed to revision of references, format, manuscript revision, and the literature search. AL contributed to literature search and measurement writing. WS contributed to data interpretation and writing the manuscript. All authors contributed to the article and approved the submitted version.

## Funding

This research was supported by internal funding #230361 and # 233263 of Massachusetts General Hospital awarded to X-JK.

## Conflict of Interest

The authors declare that the research was conducted in the absence of any commercial or financial relationships that could be construed as a potential conflict of interest.

## Publisher's Note

All claims expressed in this article are solely those of the authors and do not necessarily represent those of their affiliated organizations, or those of the publisher, the editors and the reviewers. Any product that may be evaluated in this article, or claim that may be made by its manufacturer, is not guaranteed or endorsed by the publisher.
