# Supplementary material for: Validation of Rapid Interactive Screening Test for Autism in Toddlers Using Autism Diagnostic Observation Schedule™ Second Edition in Children at High-Risk for Autism Spectrum Disorder
Source: Front Psychiatry. 2021 Oct 1;12:737890. doi: 10.3389/fpsyt.2021.737890 (PMC8517472; doi:10.3389/fpsyt.2021.737890)
Supplement: Supplementary file 1 [file Data_Sheet_1.docx]

Supplementary Material

# Supplementary Figures and Tables

**Supplementary Table 1.** RITA-T cutoff scores verses sensitivity, specificity, PPV, NPV compared with ADOS-2 and DSM-5 in 18-84 months old.

| **ADOS-2 Diagnosis (Overall)** | | | | **RITA-T Score*** | **DSM-5 Diagnosis (Overall)** | | | |
| --- | --- | --- | --- | --- | --- | --- | --- | --- |
| Sensitivity | Specificity | PPV | NPV |  | Sensitivity | Specificity | PPV | NPV |
| 1.00 | 0.14 | 0.83 | 1.00 | 6 | 1.00 | 0.08 | 0.69 | 1.00 |
| 0.97 | 0.25 | 0.83 | 0.67 | 8 | 1.00 | 0.23 | 0.72 | 1.00 |
| 0.97 | 0.50 | 0.88 | 0.80 | 9 | 0.96 | 0.31 | 0.74 | 0.80 |
| 0.91 | 0.56 | 0.88 | 0.63 | 10 | 0.89 | 0.36 | 0.73 | 0.63 |
| 0.87 | 0.75 | 0.93 | 0.60 | 12 | 0.77 | 0.31 | 0.69 | 0.40 |
| 0.80 | 0.71 | 0.92 | 0.45 | 13 | 0.72 | 0.33 | 0.69 | 0.36 |
| **0.81** | **0.89** | **0.96** | **0.57** | **14** | **0.74** | **0.50** | **0.74** | **0.50** |
| 0.72 | 0.89 | 0.96 | 0.47 | 15 | 0.63 | 0.50 | 0.71 | 0.41 |
| 0.61 | 0.88 | 0.95 | 0.37 | 16 | 0.50 | 0.46 | 0.65 | 0.32 |
| 0.57 | 1.00 | 1.00 | 0.35 | 18 | 0.44 | 0.50 | 0.65 | 0.30 |
| 0.55 | 1.00 | 1.00 | 0.36 | 19 | 0.42 | 0.54 | 0.65 | 0.32 |
| 0.52 | 1.00 | 1.00 | 0.38 | 20 | 0.39 | 0.60 | 0.65 | 0.35 |
| 0.33 | 1.00 | 1.00 | 0.26 | 21 | 0.16 | 0.50 | 0.40 | 0.22 |
| 0.32 | 1.00 | 1.00 | 0.28 | 22 | 0.15 | 0.54 | 0.40 | 0.24 |
| 0.26 | 1.00 | 1.00 | 0.26 | 24 | 0.15 | 0.69 | 0.50 | 0.29 |
| 0.19 | 1.00 | 1.00 | 0.24 | 25 | 0.15 | 0.85 | 0.67 | 0.33 |
| 0.10 | 1.00 | 1.00 | 0.21 | 26 | 0.08 | 0.92 | 0.67 | 0.32 |
| 0.07 | 1.00 | 1.00 | 0.20 | 27 | 0.04 | 0.92 | 0.50 | 0.31 |

* Identified optimal cutoff scores are highlighted in bold.

**Supplementary Table 2.** Prescreening Demographics

| **Name** | **Value** |  |
| --- | --- | --- |
| **Caregiver_teacher_concern** | No | 2 |
| **Caregiver_teacher_concern** | Yes | 33 |
| **DSM_5** | No | 10 |
| **DSM_5** | Yes | 25 |
| **MCHAT_pos** | No | 26 |
| **MCHAT_pos** | yes | 1 |
| **MCHAT_pos** | Yes | 8 |
| **Sibling.diagnosed** | No | 23 |
| **Sibling.diagnosed** | Yes | 12 |
